# Role of information and communication networks in malaria survival

**DOI:** 10.1186/1475-2875-6-136

**Published:** 2007-10-10

**Authors:** Pallab Mozumder, Achla Marathe

**Affiliations:** 1 Department of Environmental Studies and International Hurricane Research Center, University Park Campus, MARC 351, 11200 SW 8th Street, Florida International University, Miami, FL 33199, USA; 2 Network Dynamics and Simulation Science Laboratory, Virginia Bioinformatics Institute, 1880 Pratt Drive, Bldg. XV, Virginia Tech, Blacksburg, VA 24061, USA

## Abstract

**Background:**

Quite often symptoms of malaria go unrecognized or untreated. According to the Multilateral Initiative on Malaria, 70% of the malaria cases that are treated at home are mismanaged. Up to 82% of all malaria episodes in sub-Saharan Africa are treated outside the formal health sector. Fast and appropriate diagnosis and treatment of malaria is extremely important in reducing morbidity and mortality.

**Method:**

Data from 70 different countries is pooled together to construct a panel dataset of health and socio-economic variables for a time span of (1960–2004). The generalized two-stage least squares and panel data models are used to investigate the impact of information and communication network (ICN) variables on malaria death probability. The intensity of ICN is represented by the number of telephone main lines per 1,000 people and the number of television sets per 1,000 people.

**Results:**

The major finding is that the intensity of ICN is associated with reduced probability of deaths of people that are clinically identified as malaria infected. The results are robust for both indicators i.e. interpersonal and mass communication networks and for all model specifications examined.

**Conclusion:**

The results suggest that information and communication networks can substantially scale up the effectiveness of the existing resources for malaria prevention. Resources spent in preventing malaria are far less than needed. Expanded information and communication networks will widen the avenues for community based "participatory development", that encourages the use of local information, knowledge and decision making. Timely information, immediate care and collective knowledge based treatment can be extremely important in reducing child mortality and achieving the millennium development goal.

## Background

### Introduction

Every year, three million deaths are attributed to malaria, of which one-third are children. Morbidity estimates run close to 650 million just for Africa. Throughout the world five billion episodes of clinical illness occur that require anti-malarial therapy [1]. Malaria disproportionately affects poor countries, with 58% of the cases occurring in the poorest 20% of the world population [2], although 40% of the world population is at risk of malaria. Endemic malaria works through multiple channels (e.g. fertility, mortality and population growths; savings and investment; productivity and medical costs) to impede growth, development and overall human well-being. More research is needed to determine the economic and non-economic factors that limit the accessibility and use of malaria control services by the poorest [3]. Several researchers and programmes have highlighted the importance of information and communication network (ICN) facilities in controlling malaria [4,5] although no studies have investigated the relationship between ICN and malaria survival. This paper aims to fill this gap.

## Information and communication networks (ICN) and malaria prevention

More than half of the children who die of malaria die within the first 48 hours [6]. Choice between aspirin and antimalarials to treat the fever can make a difference in terms of life and death. Fast and appropriate diagnosis and treatment of malaria is extremely important in reducing child mortality and achieving two of the major millennium development goals [7]. Quite often symptoms of malaria go unrecognized or untreated [8]. According to the Multilateral Initiative on Malaria, 70% of the malaria cases that are treated at home are mismanaged. Up to 82% of all malaria episodes in sub-Saharan Africa are treated outside the formal health sector [9].

The interaction and communication between the community members can help identify the symptoms and increase the effectiveness of resources and service in place. Well-communicated information and collective decision making can lead to faster and superior home based treatment. The benefit of extensive ICN can go a long way beyond malaria prevention. It allows opening up the use of untapped resource of social capital. The authors in [10] investigate the relationships between mortality and social capital and find that communities in the US with the highest rate of civic participation (considered as a proxy of social capital) had the lowest rate of mortality from heart disease and malignant tumors. Social capital is built on shared values and norms, and is based on a sense of trust, hope, and reciprocity. Health outcomes achieved through these channels are termed as non-market behaviour. The non-market behaviour provides mutual insurance against illness and is an important determinant of the quality of health care especially in low and middle income countries [11]. Fortunately, most of the malaria-cursed developing countries are endowed with a high stock of social capital.

A variety of case studies highlighted the role of ICN facilities. Study by [12] evaluated a community-based health education programme in Kalajatha in rural India through a culturally enriched mass communication method. The findings showed that the respondents exposed to key information regarding malaria had significant positive immediate changes in knowledge and behaviour, particularly in bio-environmental measures of malaria prevention (e.g. associating clean water with anopheline breeding, releasing larvivorous fish to control malaria). The case study by in [13] finds that evidence-based participatory interventions tools that communicate key information regarding malaria are urgently required at the grassroots level. The ACCESS programme in Tanzania is another example that uses social marketing tools for providing prompt access to effective treatment for malaria at the community level [14].

Study by [5] analyses cross-country data from developing countries and shows that an increase in the stock of telecommunication infrastructure is positively correlated with an improved health status. Authors in [15] find that the communication infrastructure and information sharing are the main reasons of inefficiency in primary health care systems in rural areas in developing countries. In west African context, [16] finds that complementary investments in information, communication, health and education can significantly increase the human development index, constructed by UNDP.

The mass media and inter-personal communication channels can create a sense of urgency to react and take initiative at the early signs of malaria. Problem identification, decision-making and resolving them collectively, can increase the level of confidence of those infected, and it can pool resources and expertise at the community level to provide treatment in a timely manner. Effective communication can promote health awareness that leads to pro-active treatment of symptoms. Communication can be very effective if it is well-targeted to the groups that are most affected and most likely to act. The Roll Back Malaria (RBM) communication working group has been established to recommend appropriate communication and advocacy initiatives in this context. Given that, the working hypothesis is that ICN can be a key driver in preventing malaria deaths and the empirical analysis tends to support this hypothesis.

## Data and empirical specification

The data is compiled from two different sources to investigate the role of ICN in reducing malaria deaths. Variables pertaining to health and malaria are collected from the World Health Organization (WHO) malaria database available from the WHO web site. ICN and other relevant control variables are obtained from the World Development Indicators [17]. Data from 70 different countries is pooled together to construct a panel dataset of health and socio-economic variables for a time span of (1960–2004). The following specification is used to analyse the relationship between ICN and the likelihood of malaria deaths:

*Y*_*ij *_= *α *+ *βX*_*ij *_+ *γZ*_*ij *_+ *ε*_*ij*_

where, *Y*_*ij *_is the log of the odds of malaria death ratio for the *i*th country in the *j*th year. The malaria death ratio is calculated by dividing the number of reported malaria deaths by the number of reported malaria cases. If the dependent variable is a fraction, as in this case, it is common to use a logit transformation (log of odds) of the variable. The transformed data can be estimated by using Ordinary Least Squares technique [18]. The procedure followed here is the same to transform the dependent variable. *X*_*ij *_denotes the intensity of information and communication networks (ICN) as measured by the number of telephone main lines per 1,000 people and the number of television (TV) sets per 1,000 people. Only these two variables are considered because the data is extremely sparse for other variables such as radio and Internet connections. Telephone and TV density can capture the role of both interpersonal and mass communication networks. *Z*_*ij *_is a vector of relevant control variables to capture the heterogeneity across countries. *β *is the coefficient matrix on the intensity of information and communication networks (*X*), *α *represents the coefficient matrix on the constant term, *γ *represents the conformable vector of coefficients on *Z*, and *ε*_*ij *_represents the stochastic error term. Table 1 provides complete definitions and descriptive statistics for all the variables included in the analysis.

**Table 1:** Descriptive statistics for the dependent and independent variables

Variable	Definition	Obs.	Mean	Std dev.	Min	Max
Malaria death ratio	Log of the odds of malaria death ratio	450	0.0029	0.0063	0.00001	0.083
Telephone	Log of the number of telephone main lines per 1,000 people	4910	3.81	1.87	-2.12	6.97
TV	Log of the number of TV sets per 1,000 people	4293	4.43	1.73	-2.97	6.99
GDP	Log of per capita GDP (in constant 2,000 PPP $)	4092	8.43	1.10	6.13	11.06
Urban	Urban population (% of total)	8175	47.49	25.35	2.21	100
External resources for health	External resources for health (% of total expend. on health)	813	8.45	12.44	0	67.1
Out of pocket health Expenditure	Out-of-pocket health expend.(% of pvt. expend. on health)	834	82.05	21.59	7	100
Public health expend.	Public health expend. (as % of GDP)	832	3.58	1.88	0.25	9.73
Immunization	DPT immunization (% of children 12–23 months)	3550	73.85	25.27	1	99

The raw data shows a negative correlation between the intensity of ICN and the incidence of malaria deaths. Some of the highest malaria death rates occur in countries with the lowest availability of telephone lines and TV sets. For example, Malawi has one of the highest malaria death probability of 0.055 with a telephone density of only 4.34/1,000 people and TV density of 2.71/1,000 people. In case of Cambodia, the malaria death probability is 0.015 with the telephone density of 0.43/1,000 and TV density of 7.88/1,000 people. On the other hand, Brazil has a fairly low malaria death probability at 0.00008 with a very high telephone density of 222.91/1,000 people and TV density of 369.41/1,000 people. Similarly, in Colombia, the malaria death probability is fairly low at 0.0001 with the telephone density of 179.28/1,000 people and the TV density of 318.97/1,000 people. Although the trend between the ICN variables and malaria death probability is quite suggestive, there are countries like Somalia with low death probability (0.00008) and low density of telephone (9.84/1,000 people) and TV (14.76/1,000 people). This implies that ICN may be just one of the many factors in influencing the malaria death probability. Given the preliminary evidence of negative correlation between the malaria death probability and the ICN, the authors followed up with a more rigorous quantitative analysis to investigate the association between ICN variables and the probability of malaria deaths.

## Analysis of results

Empirical results for a variety of model specifications based on TV and telephone density are reported in Tables 2 and 3. The generalized two-stage least squares (2SLS) and panel data 2SLS models are used to investigate the impact of ICN variables on malaria death probability. The 2SLS specification is used to correct for potential endogeniety bias in ICN variables. Usually telephones and TV sets are more prevalent in urban societies and high income countries. This correlation may cause the problem of endogeniety when telephone density and TV density are used to explain the variation in malaria death probability.

**Table 2:** Generalized two stage least squares (G2SLS) estimation of parsimonious and extended models

	Model 1: Telephone		Model 2: TV	
	Stage 1	Stage 2	Stage 1	Stage 2
Dep. Var. →	Phone	Death ratio	TV	Death ratio

**Parsimonious models**				

Telephone		-0.421 (0.119) ***		
TV				-0.680 (0.164) ***
GDP	1.141 (0.108) ***		0.468 (0.106) ***	
Urban	0.020 (0.004) ***		0.030 (0.004) ***	
External res. for health	-0.008 (0.005)	-0.002 (0.009)	-0.023 (0.005)***	-0.019 (0.011)*
Out-of-pocket health expend.	0.003 (0.002)	-0.016 (0.004)***	0.013 (0.002)***	-0.006 (0.004)
Constant	-6.655 (0.935)***	-4.346 (0.641)***	-1.467 (0.928)	-3.451 (0.805)***
N	236	236	221	221
*R*^2^	0.73	0.22	0.67	0.14
F-stat	152.96 (0.000)***	10.51 (0.000)***	110.71 (0.000)***	10.51 (0.000)***

**Extended models**				

Telephone		-0.374 (0.199)*		
TV				-0.656 (0.207)***
GDP	0.687 (0.096)***		0.251 (0.111)**	
Urban	0.020 (0.003)***		0.030 (0.003)***	
External res. for health	-0.014 (0.004)	-0.004 (0.011)	-0.025 (0.005)***	-0.022 (0.012)*
Out-of-pocket Health expend.	0.005 (0.002)**	-0.018 (0.004)***	0.012 (0.002)***	-0.008 (0.005)
Public health	0.084 (0.037)**	-0.013 (0.092)	-0.015 (0.045)	-0.006 (0.090)
Immunization	0.026 (0.002)***	-0.011 (0.008)	.0142 (0.003)***	-0.009 (0.006)
Constant	-5.249 (0.755)***	-3.448 (0.555)***	-0.742 (0.883)	-2.679 (0.777)***
N	236	236	221	221
*R*^2^	0.83	0.23	0.71	0.16
F-stat	184.12 (0.000)***	13.02 (0.000)***	88.55 (0.000)***	10.71 (0.000)***

Note: ***, **, * imply significance at 1%, 5% and 10% levels, respectively; numbers in parentheses are the heteroscedasticity-corrected robust standard errors.

**Table 3:** Random effects panel data models of generalized two stage least squares (G2SLS) estimation (parsimonious and extended)

	Model 1: Telephone		Model 2: TV	
	Stage 1	Stage 2	Stage 1	Stage 2
Dep. Var. →	Phone	Death ratio	TV	Death ratio

**Parsimonious models**				

Telephone		-0.52 (0.149)***		
TV				-0.693 (0.186)***
GDP	0.965 (0.098)***		0.446 (0.127)***	
Urban	0.027 (0.004)***		0.038 (0.005)***	
External res. for health	-0.0039 (0.0029)	0.00002 (0.008)	-0.004 (0.003)	-0.002 (0.008)
Out-of-pocket health expend.	0.0029 (0.0025)	-0.013 (0.007)***	0.01 (0.003)***	-0.006 (0.007)
Constant	-5.59 (0.778)***	-4.51 (0.859)***	-1.67 (1.01)*	-3.84 (0.985)***
N	236	236	221	221
Overall *R*^2^		0.23		0.14
F-stat	0.000	0.0007	0.00	0.0006

**Extended models**				

Telephone		-0.483 (0.187)***		
TV				-0.643 (0.212)***
GDP	0.817 (0.095)***		0.381 (0.132)***	
Urban	0.026 (0.004)***		0.038 (0.005)***	
External res. for health	-0.0045 (0.002)*	0.00 (0.008)	-0.004 (0.003)	-0.002 (0.008)
Out-of-pocket health expend.	0.003 (0.002)	-0.013 (0.007)***	0.01 (0.003)***	-0.006 (0.007)
Public health	0.023 (0.033)	0.003 (0.103)	0.019 (0.046)	0.009 (0.108)
Immunization	0.011 (0.002)***	-0.005 (0.006)	0.004 (0.002)***	-0.006 (0.006)
Constant	-5.3 (0.722)***	-4.22 (0.868)***	-1.53 (1.01)	-3.52 (1.01)***
N	236	236	221	221
Overall *R*^2^		0.23		0.16
F-stat	0.00	0.0008	0.00	0.0012

Note: ***, **, * imply significance at 1%, 5% and 10% levels, respectively; numbers in parentheses are the heteroscedasticity-corrected robust standard errors.

In the generalized 2SLS regression, the first stage is used to create an exogenous variable which is then used as an independent variable in the second stage for consistent estimates. In this study, the first stage involves regressing telephone/TV density on instrumental variables i.e. urbanization (measured by proportion of urban population) and economic development (measured by per capita GDP). The instrumental variables are highly correlated with the endogenous explanatory variable i.e. the telephone and TV density but have no strong correlation with the outcome variable i.e. the malaria death ratio [19]. The pairwise correlations are available from the authors upon request. The first stage results in fitted values of telephone density and TV density that have been adjusted for urbanization and economic development. In the second stage, malaria death probability is regressed on the newly created fitted variables and other explanatory variables. The results show that even after accounting for the potential endogeniety through valid instruments, the information and communication networks (measured by the number of main telephone lines and TV sets per 1,000 people) show a significant association with reduced probability of malaria deaths at a global level.

Table 2 presents the generalized 2SLS estimates of the parsimonious and extended models. Table 3 shows the random effects panel data models. The panel data models are selected to correct for possible country specific temporal autocorrelation across years. To check robustness of the findings some additional control variables (e.g. public health expenditure and immunization) are added in the extended models. Results are consistent for both interpersonal and mass communication channels (i.e., telephone lines and TV sets) and findings are robust across different model specifications. The estimated coefficients on ICN variables in all models reported in Tables 2 and 3 indicate a highly significant negative relationship, implying reduced likelihoods of malaria deaths with increasing intensity of ICN. Furthermore, the impact of TV density is much higher compared to telephone density across all models implying that public and mass media has a higher influence on establishing effective ICN outreach.

In the two stage least squares estimation, it is evident (from the first stage) that more telephones and TV sets are associated with people who earn more money, live in urban areas and have better track record of immunization. In addition, people with better ICN facilities tend to have more out-of-pocket health expenditure and receive less external resources for health in the form of development aid. The second stage results show that even after controlling for all these possible factors, the density of telephones and TV sets is significantly related to the malaria death probability. Results regarding relevant control variables in the second stage show that external resources for health also contribute significantly in reducing the malaria death probability. Higher proportion of out-of-pocket health expenditure is also found to reduce the likelihood of malaria deaths.

**Figure 1:**
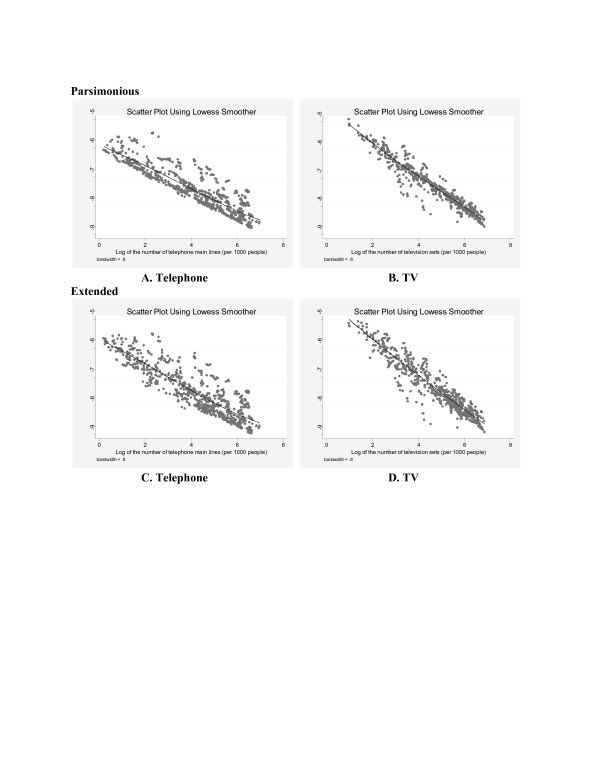
Generalized two-stage least squares estimation.

**Figure 2:**
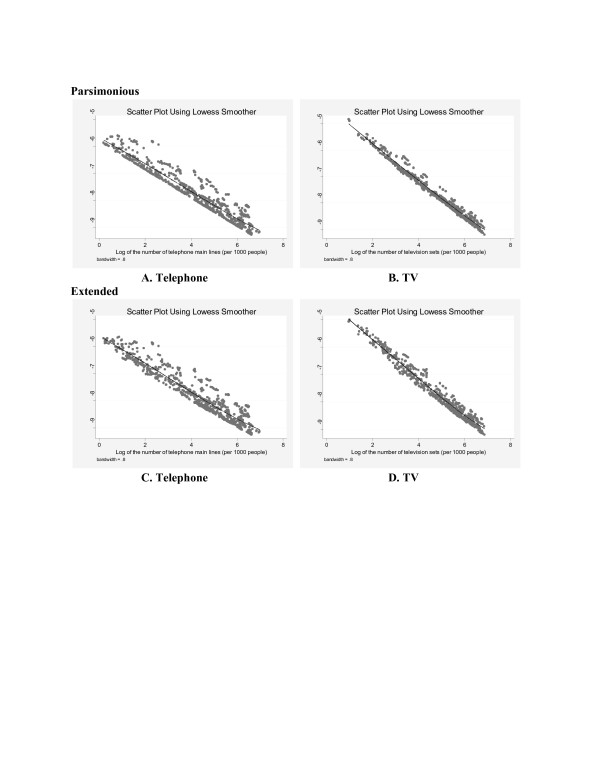
Panel data two-stage least squares estimation.

The intensity of ICN is found to be a significant covariate in explaining the likelihood of deaths of people that are clinically identified as malaria infected. The results are robust for both indicators (i.e. interpersonal and mass communication networks) and for all model specifications examined. Figure 1 shows scatter plots (using lowess smoother) of the fitted values of malaria death probability against telephone density and TV density for both parsimonious and extended models. Figure 2 shows the same plots for panel data models. Two important observations are: (i) TV density shows a stronger negative association with the probability of malaria deaths compared to telephone density (viewed by relatively steeper line for TV density) and (ii) the fitted data points are more scattered in all four graphs of generalized models (in Figure 1) compared to the graphs of panel data models (in Figure 2). This suggests that panel data models which take into account the country specific temporal autocorrelation, provide a better fit of the data analysed.

Note that the empirical analysis is founded on an estimated reduced form equation based on the data available across countries. Therefore, these results should be considered as an evidence of suggestive trend instead of any causal inference. Carefully designed randomized experiments (where investigators have better control over the confounding factors) can explore causal relationships between the ICN variables and malaria incidences.

## Summary and discussion

This study uses telephone density and TV density as two major ICN variables to investigate its impact in reducing malaria deaths. The cross country analysis shows that the ICN density as measured by the telephones and TV sets per 1,000 people has a significant correlation with reduced probability of malaria deaths. Telephones, an inter-personal means of communication, connects individuals to a bigger social network and facilitates rapid diagnosis and treatment, whereas TV, a mass media communication means, can provide disease awareness among the population, encourage people to adopt healthy behaviour and communicate risk mitigation measures.

In preventing malaria, ICN may not have a direct impact similar to malaria drugs but it can certainly increase the effectiveness of the intervention strategies and resources indirectly. ICN can speed up the delivery of services and provide access to crucial health information [20]. Access to information and knowledge allows the community members to participate in opportunities and activities related to their own development [21].

There have been concerns raised by researchers that more availability of telephone lines and Internet connections will not address the fundamental problems in developing countries which arise mainly because of unequal distribution of wealth and inequitable access to technology [22]. A successful effort of malaria prevention would have to promote access of ICN facilities to the disadvantaged and deprived classes in the society. Recently there have been some exciting innovations to address the needs of the disadvantaged groups in the society. For instance, the cellular phone operations by Grameen Telecom (GTC) in Bangladesh, where telephone density is one of the lowest in the world, has become a very successful venture [23]. Another initiative, Grameen Communications (GC) has developed a number of multipurpose tele-centers in the rural areas that provide villagers access to information, communication, services and education relating to information technology [23].

Grameen Foundation, USA is striving to expand similar programmes in Philippines, Uganda and a few other African countries. A primary objective of the GC is to provide services to the poor people who are identified as "most novice high end users" and have been largely left out of the information technology revolution. GC is planning to install graphical interfaces and interactive multimedia objects to provide critical health care information to the villagers who are largely illiterate. GC is using these tools to increase health awareness on infectious diseases and to train rural doctors and health workers. These initiatives also open up avenues for "social entrepreneurship" [24] in the private sector and the corporate world who intend to participate in the fight against malaria by lending their upper hand in resources and know-how to spur information and communication technology.

Even in the 21st century there are many countries with very poor ICN coverage. These countries happen to be some of the poorest countries in the world. Grameen type expansion of ICN needs to be implemented at a global scale to fight against poverty and malaria and to achieve the millennium development goal [25,26]. ICN facilities will foster networking within, between and beyond communities and add to the stock of social capital that can be used to scale up the productivity of limited resources to fight against malaria [27]. It has been shown that neighbourhoods characteristics that are conducive to social communication and networking contribute to the formation of social capital [28].

Expanded information and communication networks also widen the avenues for community based "participatory development", an approach that encourages the use of local information, knowledge and decision making to define and steer the nature of an intervention [29]. Participation of local communities, communication with the marginalized, relationship and trust building empower communities and help them engage in collective action [30-32]. Participatory communication environment not only permits expression of diverse ideas on societal concerns but also harnesses the strengths of traditional media [33]. Work by [34] identifies lack of community participation and decision making as some of the major constraints in malaria interventions.

The communities should not be perceived only as consumers of malaria preventing initiatives, but also as active participating input in these pursuits. Collaborative effort, shared responsibility and ownership of ideas yield mutual benefits to the participants and vastly scale up the effectiveness of the malaria intervention efforts. Integration of community based distribution of anti-malarials and malaria information and education has been shown to reduce under-five age mortality by 41% in one Ethiopian programme [35]. Communication is critical to changing household practices, increasing the demand for malaria preventing services and products, improving home based care, and mobilizing communities for malaria control. Technological devices (e.g. telephones, TVs) multiply the role of key information as they provide more efficient modes of communication i.e. reach more people across a larger space in a shorter time. While individual communication media such as telephones can be instrumental resources in critical conditions, mass media such as TVs can be used to promote malaria drugs, ITNs, appropriate treatment options etc. [4]. Resources spent in preventing malaria are far less than needed. An outreach programme through effective ICN should not be considered as a substitute to the key inputs of malaria prevention but as a complement to scale up the effectiveness of these inputs in the fight against malaria. ICN is only one aspect of an effective multi-prong approach in addressing the malaria problem.

## Competing interests

The author(s) declare that they have no competing interests.
